# A case of breast cancer with extensive colon metastasis

**DOI:** 10.1002/deo2.189

**Published:** 2022-11-27

**Authors:** Jin Imai, Toru Hanamura, Aya Kawanishi, Takashi Ueda, Yusuke Mishima, Ayano Ito, Yoshihiro Shirataki, Masashi Morimachi, Toshio Kodama, Hirohiko Sato, Motoki Kaneko, Masaya Sano, Erika Teramura, Makiko Monma, Shingo Tsuda, Kota Tsuruya, Hajime Mizukami, Yoshitaka Arase, Mia Fujisawa, Saya Miyahara, Naoya Nakamura, Takayoshi Suzuki, Masashi Matsushima, Hidekazu Suzuki, Shinji Takashimizu, Tatehiro Kagawa, Yasuhiro Nishizaki

**Affiliations:** ^1^ Department of Clinical Health Science Tokai University School of Medicine Isehara Kanagawa Japan; ^2^ Department of Breast and Endocrine Surgery Tokau University School of Medicine Isehara Kanagawa Japan; ^3^ Department of Internal Medicine, Division of Gastroenterology Tokai University School of Medicine Isehara Kanagawa Japan; ^4^ Department of Pathology Tokai University School of Medicine Isehara Kanagawa Japan

**Keywords:** breast cancer, colon, lobular carcinoma, malignancy, metastasis

## Abstract

Breast cancer is one of the most common malignancies in women worldwide. Although most breast cancers are curable, in cases of metastasis, many are often found in the lungs, bones, liver, and central nervous system; however, metastasis to the gastrointestinal tract is rare. Invasive lobular carcinoma, which represents only 5%–10% of breast cancers, has a higher risk of metastasis to the gastrointestinal tract than invasive ductal carcinoma. Here, we report a rare case of gastrointestinal metastasis of invasive lobular carcinoma that spread extensively to the colonic mucosa. Given the improved survival rates of breast cancer patients with current treatments, many rarer metastatic diseases, including gastrointestinal metastases, are likely to be increased in the future.

## INTRODUCTION

Breast cancer is one of the most common malignancies among women worldwide with an estimated 1,300,000 new cases and 465,000 deaths annually.^1^, ^2^ The bones, lungs, liver, and central nervous system are well known as the primary target sites of breast cancer metastasis; however, metastasis to the gastrointestinal tract is very rare.^3^ Invasive lobular carcinoma, which represents only 5%–10% of breast cancers, has a higher risk of metastasis to the gastrointestinal tract than invasive ductal carcinoma.^4^ In this report, we describe a rare case of Invasive lobular carcinoma with extensive metastasis to the colon.

## CASE REPORT

**FIGURE 1:**
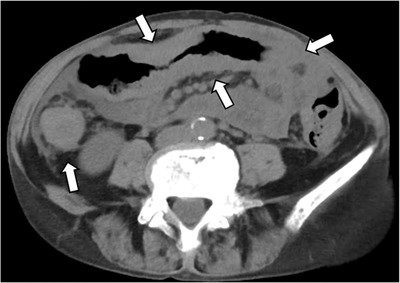
The computed tomography scan showed extensive wall thickening of the transverse colon to the ascending colon.

**FIGURE 2:**
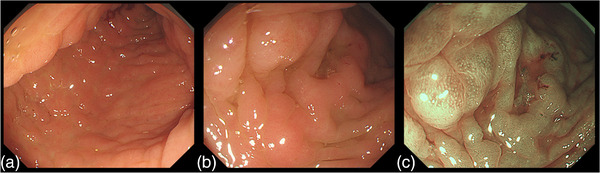
(a,b) Colonoscopy indicated sclerotic changes in the colon wall and loss of vascular permeability, which were seen extending continuously from the ascending colon to the transverse colon. (c) Dilated crypts were observed in narrow‐band imaging.

**FIGURE 3:**
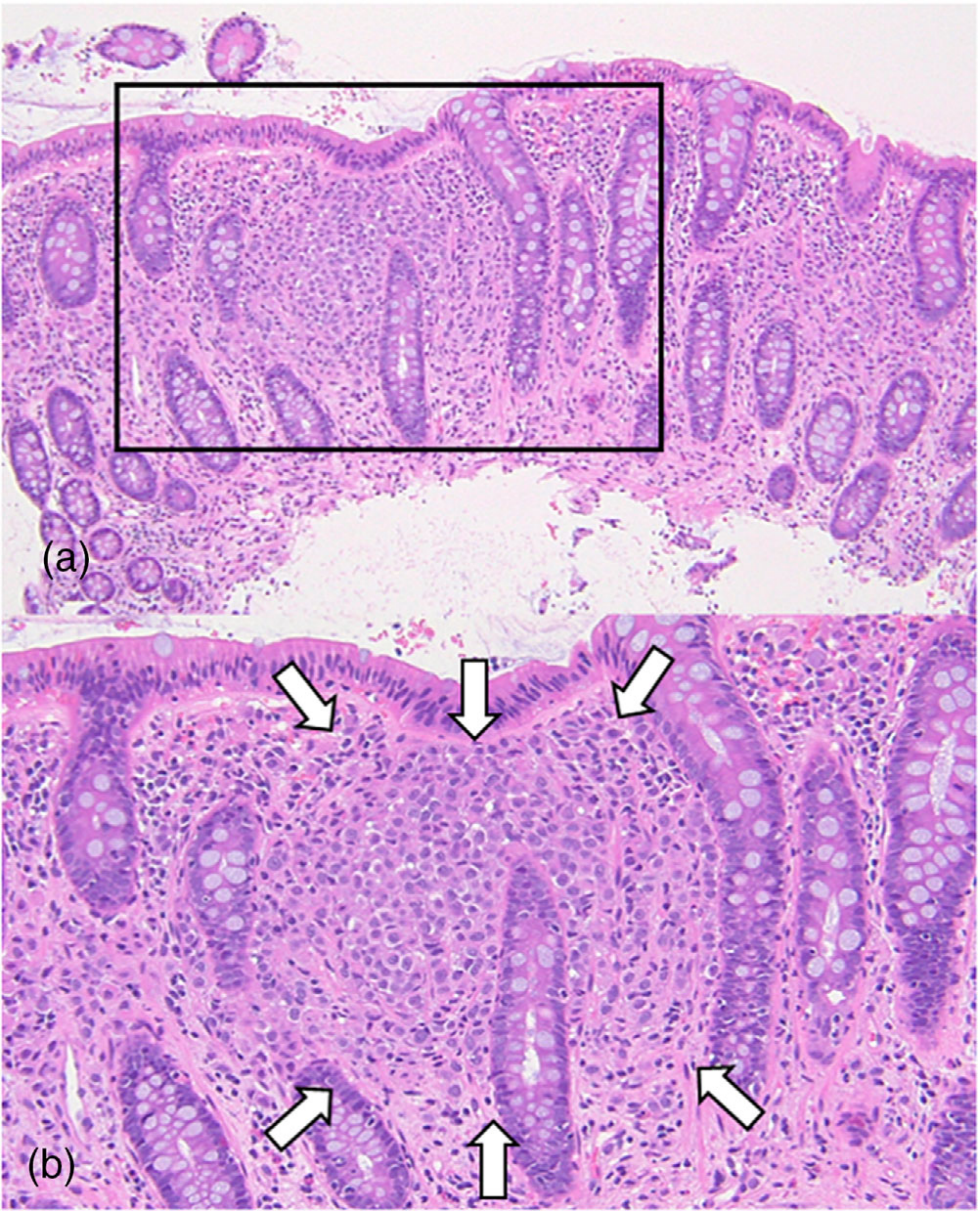
Hematoxylin and eosin staining of the colonic biopsy samples indicated the infiltration of tumor cells in the lamina propria. (a) x100 (b) x400

**FIGURE 4:**
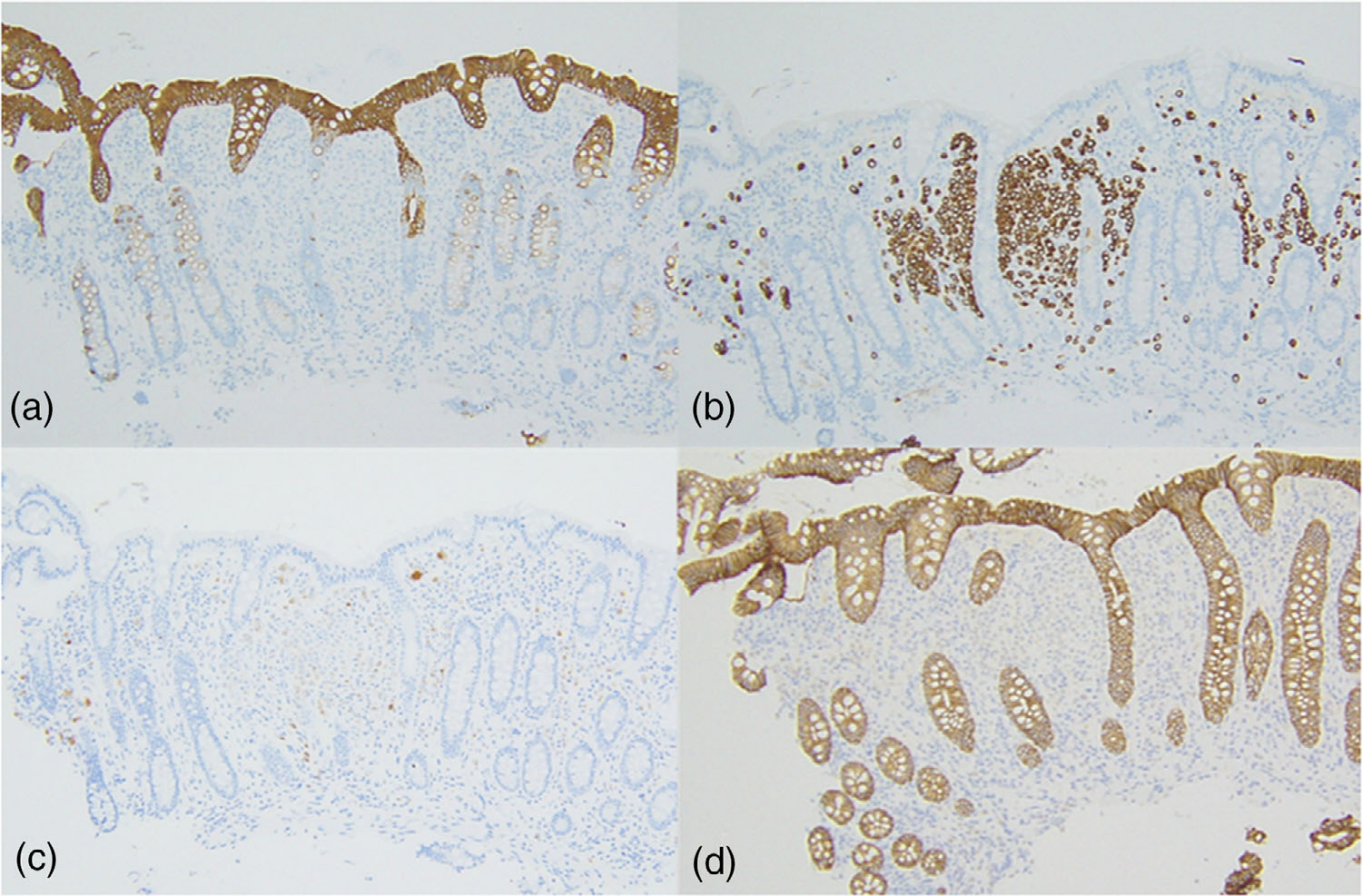
Immunohistochemical staining of the colonic biopsy samples were (a) CK20 negative, (b) CK7 positive, (c) GCDFP15 positive, and (d) E‐cadherin negative (x100).

A 72‐year‐old woman underwent a neck computed tomography scan at the time of a traffic accident, indicating a bone translucency image. Malignancy screening revealed right breast cancer with bone metastases to the spine, ribs, and pelvis. The histopathology of breast cancer was invasive lobular carcinoma. Her tumor stage was diagnosed with stage IV, and the patient was started on fulvestrant treatment. Two years after starting treatment, a follow‐up computed tomography scan showed extensive wall thickening from the transverse colon to the ascending colon (Figure 1). Sclerotic changes to the colon wall and loss of vascular permeability were seen via colonoscopy extending continuously from the ascending colon to the transverse colon (Figure 2a,b). Dilated crypts could also be seen with narrow‐band imaging (Figure 2c). Hematoxylin and eosin staining of colonic biopsy samples indicated infiltration of tumor cells in the lamina propria (Figure 3a,b). Immunohistochemical staining of colonic biopsy samples showed them to be CK20 negative, CK7 positive, GCDFP15 focally positive, E‐cadherin negative, Mammaglobin negative and ER positive (Figure 4a–d and Figure S1a,b), which was the same phenotype as the primary cancer lesion. She was diagnosed with extensive colon metastasis of the breast invasive lobular carcinoma.

## DISCUSSION

Mammary malignancies exhibit a distinct pattern from systemic metastasis. Invasive ductal carcinoma is usually associated with liver, lung, and brain metastases, whereas invasive lobular carcinoma, representing only 5%–10% of all breast cancer cases,^4^, ^5^ frequently affects the gynecological organs, peritoneum, retroperitoneum, and gastrointestinal (GI) tract.^3^ The stomach and small intestine are the most frequent sites of metastatic lesions in the GI tract; colonic and rectal metastases are very rare.^6^ The reason for this increased risk of metastasis within the GI tract is not clear, but may be related to the loss of E‐cadherin, a cell‐cell adhesion molecule, in these cancer types.^7^ In the present case, we confirmed that E‐cadherin did not stain positive within the tumor cells by immunohistochemical.

The extensive lateral extension of the colonic mucosa was found in the present case, although it is very rare among breast cancers with GI metastasis. Maharajh et al. reported a similar case of extensive metastasis that showed diffuse thickening of the terminal ileum.^8^ Rubin et al. reported a sigmoid structure due to breast cancer metastasis.^9^ Interestingly, these cases were difficult to differentiate from Crohn's disease. Although the present case lacked longitudinal ulcers or other inflammatory features and could be differentiated from Crohn's disease, it is important to keep in mind that this type of metastasis is possible in invasive lobular carcinoma. Moreover, one histological feature of colonic metastasis from invasive lobular carcinoma is mainly its existence in the submucosal and muscles layer.^10^ Therefore, deep‐sampling biopsies during colonoscopy are often required, and it is also important to inform the pathologist of the possibility of breast cancer metastasis. In the present case, the tumor cells were detected in two of the four biopsies, because fortunately, the infiltration of tumor cells was invaded into the mucosal layer as Figure 3. Although we performed random biopsies, we believe that a detailed endoscopic picture of the mucosal invasion features will contribute to the identification of lobular carcinoma in the future.

Finally, this patient had no gastrointestinal symptoms, and imaging studies coincidentally led to the diagnosis of metastases. However, the above two previously reported cases^8^, ^9^ were complicated by obstructive symptoms. Moreover, Nazareno et al. reported that gastrointestinal metastases were detected more than 10 years after the first diagnosis^4^; therefore, the possibility of gastrointestinal metastasis should be considered, especially in patients with primary breast invasive lobular carcinoma who complain of gastrointestinal symptoms, regardless of the duration of the post‐diagnosis period.

## CONFLICT OF INTEREST

The authors declare that they have no conflict of interest.

## INFORMED CONSENT

This case report was performed in accordance with the World Medical Association Declaration of Helsinki. Informed consent was obtained from the patient for the publication of this case report and the accompanying images.

## Supporting information


**Figure S1** Immunohistochemical staining of the colonic biopsy samples; Mammaglobin and ER.

## References

[1] Kamangar F , Dores GM , Anderson WF . Patterns of cancer incidence, mortality, and prevalence across five continents: Defining priorities to reduce cancer disparities in different geographic regions of the world. J Clin Oncol 2006; 24: 2137–50. 16682732 10.1200/JCO.2005.05.2308

[2] Ha R , Chow D , Mango V *et al*. Have we given up on breast cancer metastasis? Global trends in breast cancer metastasis research productivity. Breast J 2015; 21: 442–4. 25982155 10.1111/tbj.12436

[3] Ciulla A , Castronovo G , Tomasello G *et al*. Gastric metastases originating from occult breast lobular carcinoma: Diagnostic and therapeutic problems. World J Surg Oncol 2008; 6: 78. 18652707 10.1186/1477-7819-6-78 PMC2525652

[4] Nazareno J , Taves D , Preiksaitis HG . Metastatic breast cancer to the gastrointestinal tract: A case series and review of the literature. World J Gastroenterol 2006; 12: 6219–24. 17036400 10.3748/wjg.v12.i38.6219 PMC4088122

[5] Thomas M , Kelly ED , Abraham J *et al*. Invasive lobular breast cancer: A review of pathogenesis, diagnosis, management, and future directions of early stage disease. Semin Oncol 2019; 46: 121–32. 31239068 10.1053/j.seminoncol.2019.03.002

[6] Falco G , Mele S , Zizzo M *et al*. Colonic metastasis from breast carcinoma detection by CESM and PET/CT: A case report. Medicine 2018; 97: e10888. 29794798 10.1097/MD.0000000000010888 PMC6392653

[7] Lehr HA , Folpe A , Yaziji H *et al*. Cytokeratin 8 immunostaining pattern and E‐cadherin expression distinguish lobular from ductal breast carcinoma. Am J Clin Pathol 2000; 114: 190–6. 10941333 10.1309/CPUX-KWEH-7B26-YE19

[8] Maharajh S , Capildeo K , Barrow M *et al*. Case report of metastatic breast cancer mimicking ileal Crohn's disease. Int J Surg Case Rep 2021; 87: 106408. 34534815 10.1016/j.ijscr.2021.106408 PMC8449070

[9] Rubin JCKE , Wong U , Cross RJ , Bafford A , Patil S . Case of invasive metastatic breast cancer mimicking Crohn's disease: 2065 Off J Am Coll Gastroenterol ACG 2018; 113: S1176.

[10] Tsujimura K , Teruya T , Kiyuna M *et al*. Colonic metastasis from breast carcinoma: A case report. World J Surg Oncol 2017; 15: 124. 28679405 10.1186/s12957-017-1193-5 PMC5498884

