# Supplementary material for: A case of breast cancer with extensive colon metastasis
Source: DEN Open. 2022 Nov 27;3(1):e189. doi: 10.1002/deo2.189 (PMC9702338; doi:10.1002/deo2.189)
Supplement: Supplementary file 1 — Figure S1 Immunohistochemical staining of the colonic biopsy samples; Mammaglobin and ER. [file DEO2-3-e189-s001.pdf]

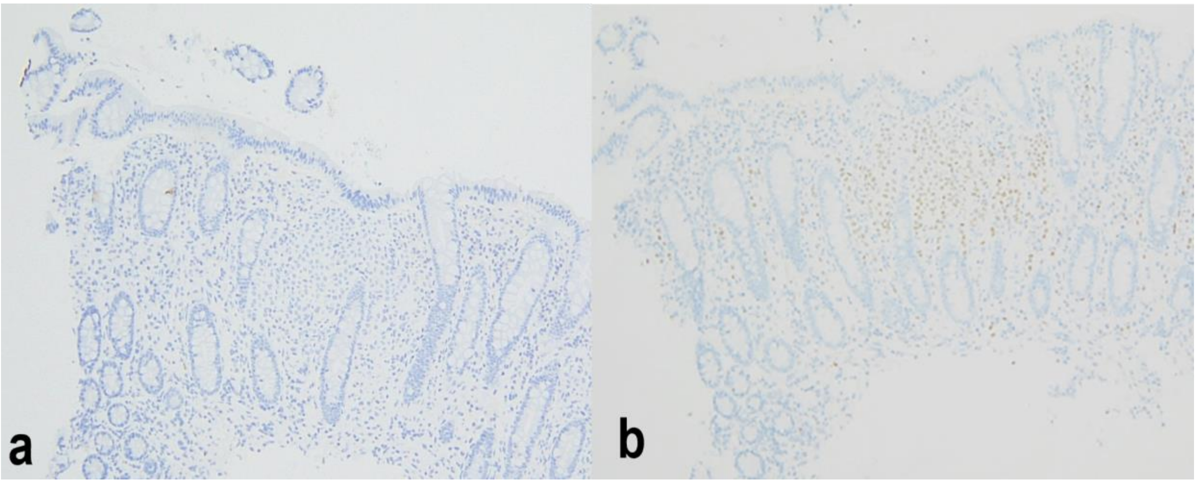

Supplement Fig.1. Immunohistochemical (IHC) staining of the colonic biopsy samples were (a) Mammaglobin negative, (b) ER positive. (x100)
